# Fast Screening of Tyrosinase Inhibitors in *Coreopsis tinctoria* Nutt. by Ligand Fishing Based on Paper-Immobilized Tyrosinase

**DOI:** 10.3390/molecules29174018

**Published:** 2024-08-25

**Authors:** Ayzohra Ablat, Ming-Jie Li, Xiao-Rui Zhai, Yuan Wang, Xiao-Lin Bai, Peng Shu, Xun Liao

**Affiliations:** 1 Chengdu Institute of Biology, Chinese Academy of Sciences, Chengdu 610041, China; ayi@cib.ac.cn (A.A.); zhaixr@cib.ac.cn (X.-R.Z.); baixl@cib.ac.cn (X.-L.B.); 2 University of Chinese Academy of Sciences, Beijing 100049, China; 3 HBN Research Institute and Biological Laboratory, Shenzhen Hujia Technology Co., Ltd., Shenzhen 518000, China; limingjie@hbn.cn (M.-J.L.); wangyuan@hbn.cn (Y.W.)

**Keywords:** *Coreopsis tinctoria*, snow chrysanthemum, ligand fishing, tyrosinase inhibitors

## Abstract

*Coreopsis tinctoria* Nutt. is an important medicinal plant in traditional Uyghur medicine. The skin-lightening potential of the flower has been recognized recently; however, the active compounds responsible for that are not clear. In this work, tyrosinase, a target protein for regulating melanin synthesis, was immobilized on the Whatman paper for the first time to screen skin-lightening compounds present in the flower. Quercetagetin-7-*O*-glucoside (**1**), marein (**2**), and okanin (**3**) were found to be the enzyme inhibitors. The IC_50_ values of quercetagetin-7-*O*-glucoside (**1**) and okanin (**3**) were 79.06 ± 1.08 μM and 30.25 ± 1.11 μM, respectively, which is smaller than 100.21 ± 0.11 μM of the positive control kojic acid. Enzyme kinetic analysis and molecular docking were carried out to investigate their inhibition mechanism. Although marein (**2**) showed a weak inhibition effect in vitro, it inhibited the intracellular tyrosinase activity and diminished melanin production in melanoma B16 cells as did the other two inhibitors. The paper-based ligand fishing method developed in this work makes it effective to quickly screen tyrosinase inhibitors from natural products. This is the first report on the tyrosinase inhibitory effect of those three compounds, showing the promising potential of *Coreopsis tinctoria* for the development of herbal skin-lightening products.

## 1. Introduction

*Coreopsis tinctoria* Nutt., an annual herbaceous plant classified within the Compositae family, is native to North America and widely distributed in China. The wild plant is mainly found in Xinjiang Uyghur Autonomous Region, growing above the snowline of around 3000 m in the Kunlun Mountains, thus it is commonly called Kunlun snow chrysanthemum. The unique habitat is characterized by a cold and humid climate with thin air, and the yield of this plant is very low. Owing to its great health and medicinal value, this plant has been widely cultivated as an economic crop in plain areas across China [1].

The flower of *C. tinctoria* is used to make precious tea, and it has a long-standing history of utilization in traditional Uyghur medicine for the treatment of cardiovascular diseases [2]. It is renowned for its rich contents of vitamins, flavonoids, and phenolics, which are associated with a variety of health benefits [3]. It has been reported that the flower extract exhibits multiple biological activities, such as antihyperglycemic, antihypertensive, antioxidative, anticancer, antimicrobial, and anti-inflammatory activities [4,5]. Flavonoids are the main active ingredients subjected to thorough investigation in the extract, which are beneficial for managing conditions such as cancer, neurological disorders, cardiovascular diseases, and disturbances in glucose and lipid metabolism [6,7,8]. Recently, the flower of *C. tinctoria* was found to have the potential for skin lightening, which might bring new prospects for the application of this plant [9]. However, investigations of the active components responsible for this effect are notably scarce.

Tyrosinase (TYR) is a copper-containing polyphenol oxidase that plays a critical role in the regulation of melanin formation [10,11]. It catalyzes the hydroxylation and subsequent oxidation of monophenol units into orthoquinone, which is essential for determining the pigmentation of hair and skin [12]. In humans, melanin serves as a protective agent against ultraviolet radiation damage. However, excessive melanin can lead to abnormal pigmentary disorders such as melasma, freckles, post-inflammatory hyperpigmentation, and pigmented acne scars, and may even contribute to the formation of malignant melanoma [13]. Therefore, TYR inhibitors are regarded as a potential solution for the diseases caused by disorders of tyrosinase and melanin biosynthesis. In the meantime, TYR inhibitors from natural sources have attracted intensive attention, for example, arbutin, resveratrol, and kojic acid [14]. However, most of them exhibited disadvantages of cytotoxicity, insolubility, and weak cutaneous absorption, which constrained their further utilization [15,16].

Fast screening of active compounds from complex herbal extracts is challenging. Various affinity-based techniques have been developed to find enzyme inhibitors such as affinity chromatography [17], affinity ultrafiltration [18], and affinity solid-phase extraction (also called ligand fishing) [19]. Among them, ligand fishing has been recognized as one of the most efficient strategies [20]. By immobilizing TYR on magnetic nanoparticles used as the ligand fishing baits, our group discovered some new TYR inhibitors, e.g., baicalin, baicalein, wogonin, and oroxylin A from *Scutellaria baicalensis* [21], and caffeic acid, rosmarinic acid from Spica Prunellae [22]. Although this method exhibits high sensitivity, it requires stringent reaction conditions and has a prolonged experimental duration. Apart from magnetic nanoparticles, a variety of solid phases like nanotubes, hollow fibers, and microfluidic chips have been used to immobilize target proteins in ligand fishing [23]. However, those methods encounter the disadvantages of expensive target proteins, tedious protein immobilization, and usually off-targeted results. To solve these problems, the filtration paper has attracted attention as the solid phase for protein immobilization due to its low cost and easiness to be surface-modified [24]. For instance, the α-glucosidase-functionalized filter paper was successfully employed for inhibitor screening from 14 kinds of traditional medicinal plant extracts [25].

In this study, we immobilized tyrosinase onto dopamine (DA)-modified Whatman cellulose chromatography paper (CP) for the first time (CP@DA@TYR) and applied it in the screening of tyrosinase inhibitors from the flowers of *C. tinctoria*. The ligands were identified using UFLC-MS in combination with the comparison to the standard compounds, and their TYR inhibitory activities were tested by an enzymatic inhibition assay and the melanoma B16 cells. The inhibition types and interaction mechanisms of these compounds against TYR were elucidated by enzyme kinetic analyses and molecular docking.

## 2. Results and Discussion

### 2.1. Characterization of CP@DA@TYR

**Figure 1:**
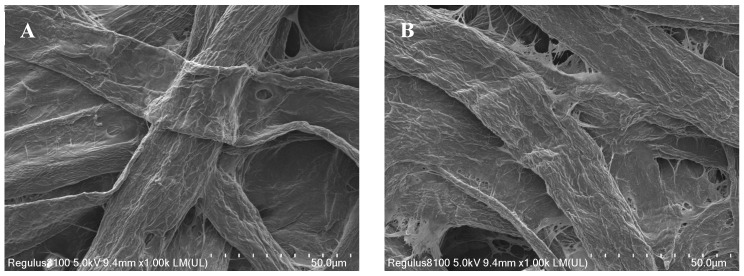
Scanning electron microscopy (SEM) images of (**A**) cellulose chromatography paper and (**B**) CP@DA@TYR.

The TYR was immobilized on cellulose chromatography paper for the first time in this study. The surface morphology of CP@DA@TYR was compared with cellulose chromatography paper by scanning electron microscopy (SEM). As shown in Figure 1 and Figure S2, the interlacing borders between the fibrous pores are clear for cellulose chromatography paper, while there are obvious flocculent objects adsorbed on the surface of the paper, which indicates the successful immobilization of TYR on the cellulose chromatography paper.

The enzyme immobilized on the cellulose chromatography paper was determined to be 2.65 µg/mm^2^.

### 2.2. Selection of the Buffer System

**Figure 2:**
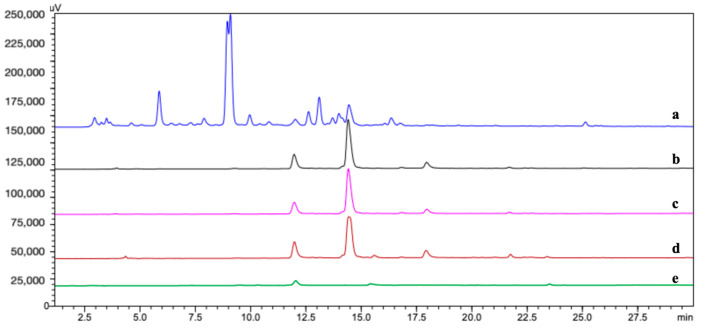
The influence of different buffer solvent systems on fishing results. (**a**) Extraction of *C. tinctoria* (S0); (**b**) CP@DA@TYR-S5 (compounds fished by CP@DA@TYR) in pH7.8 Tris-HCl; (**c**) CP@DA@TYR-S5 in pH6.0 PBS; (**d**) CP@DA@TYR-S5 in ultrapure water; and (**e**) CP@DA-BS5 (blank control: compounds fished by CP@DA).

The type of buffer system is an important parameter influencing the enzymatic reaction as well as the outcome of ligand fishing. We tested the effects of different buffers at a pH range of 6–8 on the fishing ability of CP@DA@TYR. As demonstrated in Figure 2, the type of buffer in this pH range had little influence on the fishing ability of CP@DA@TYR. It is worth noting that TYR immobilized on cellulose paper exhibited the same fishing efficiency in pure water as in the other two buffer systems, making it a much more convenient fishing bait than traditional baits needing buffer systems [26].

### 2.3. Identification of the TYR Ligands

**Figure 3:**
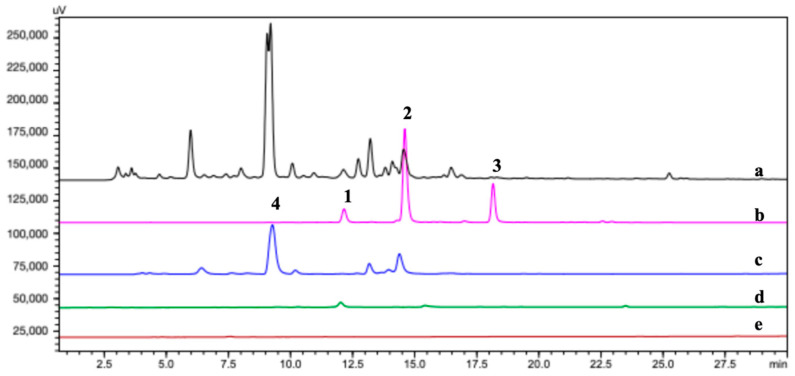
HPLC chromatograms (280 nm) of (**a**) S0, (**b**) CP@DA@TYR-S5, (**c**) MNPs@TYR-S5, (**d**) CP@DA-BS5, and (**e**) MNPs-BS5.

In a preliminary experiment, we used both magnetic nanoparticle-immobilized TYR (MNPs@TYR) and cellular chromatography paper-immobilized TYR (CP@DA@TYR) to “fish” the ligands from crude extract of *C. tinctoria* flowers. The outcomes of the two methods are significantly different as shown in Figure 3. The major compound retrieved by MNPs@TYR was chlorogenic acid (**4**) [27], whereas compound **2** was the predominant one extracted by CP@DA@TYR. The reason for this interesting difference might lie in the discrepancy between the immobilization methods and the support materials [28]. For the CP@ DA@TYR, polydopamine plays a vital role in the binding of the enzyme. Polydopamine is a versatile material that can effectively adhere to various substrate surfaces [29], and it is featured by quinone and phenolic hydroxyl groups facilitating Schiff base and Michael addition reactions with protein macromolecules, which can be used to immobilize proteins in a milder way than other covalent binding methods [30,31]. The in vitro TYR inhibitory activities of the mixture of fished compounds (S5s) exhibited significant differences, as shown in Table S1.

The HPLC chromatogram of S5 of the crude extraction (Figure 3) showed that three compounds including **1** (Rt = 12.1 min), **2** (Rt = 14.2 min), and **3** (Rt = 18.2 min) were fished out using the CP@DA@TYR. By comparison, the blank control CP@DA did not fish out any components, further revealing that those three ligands had specific binding with the immobilized TYR.

**Figure 4:**
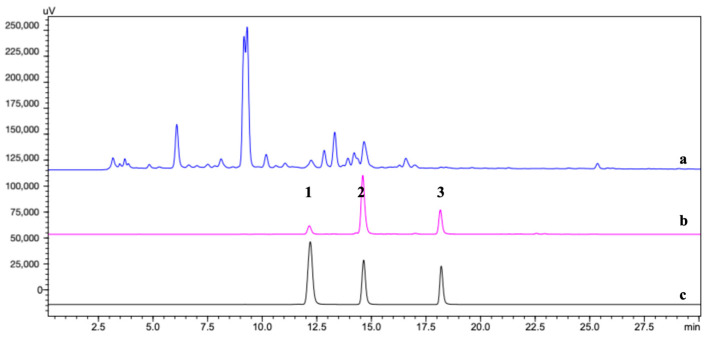

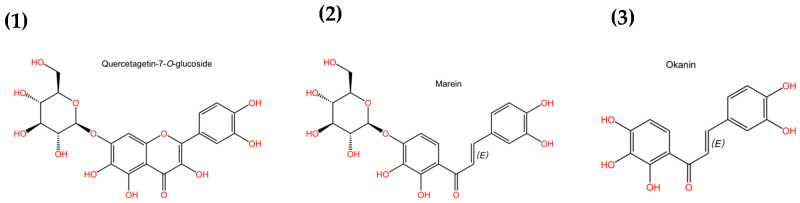
HPLC chromatograms of ligand fishing of *C. tinctoria*. (**a**) S0, (**b**) S5, and (**c**) standard mixture of quercetagetin-7-*O*-glucoside, marein, and okanin. Chemical structures of (**1**) quercetagetin-7-*O*-glucoside, (**2**) marein, and (**3**) okanin.

Structures of the obtained compounds were identified by ultra-performance liquid chromatography-mass spectrometry (UPLC-MS). As shown in Figure S3, compound **1** produced the pseudo-molecular ions of *m*/*z* 481.09776 [M + H]^+^, *m*/*z* 503.07959 [M + Na]^+^, and *m*/*z* 479.08387 [M−H]^−^, and a daughter ion of *m*/*z* 319.04495 [M + H − C_6_H_10_O_5_]^+^; compound **2** yielded the pseudo-molecular ions of *m*/*z* 451.12373 [M + H]^+^, *m*/*z* 473.10579 [M + Na]^+^, and *m*/*z* 449.10977 [M − H]^−^,and a daughter ion of *m*/*z* 289.07111 [M + H − C_6_H_10_O_5_]^+^; compound **3** gave the pseudo-molecular ions of *m*/*z* 289.07103 [M + H]^+^, *m*/*z* 311.05276 [M + Na]^+^, and *m*/*z* 287.05668 [M − H]^−^. Finally, by comparing MS and HPLC data to those of the reference standards and reported in the literature, compounds **1**–**3** were identified as quercetagetin-7-*O*-glucoside, marein, and okanin as shown in Figure 4.

### 2.4. Quantification of the TYR Ligands

Linear calibration curves of concentration versus the peak area were established using HPLC for compounds **1**–**3**, with calibration ranges of 0.6–10, 5–80, and 0.3–10 ug/mL with R^2^ > 0.999. Consequently, based on the peak area of the analyte compounds in S0, the contents of compounds **1**–**3** in the dry plant were calculated to be 6.03 ± 0.98, 45.80 ± 1.24, and 8.24 ± 0.76 mg/g, respectively.

### 2.5. TYR Inhibitory Activity

The outcomes of the in vitro TYR inhibitory activity showed that marein (**2**) possesses weak inhibition of 29.38% at 25 μM, while compounds **1** and **3** exhibited a potent inhibitory effect with IC_50_ values of 79.06 ± 1.08 and 30.25 ± 1.11 μM, both of which are lower than the IC_50_ for kojic acid (IC_50_ = 100.21 ± 0.01 μM). This is the first report on the TYR inhibitory activity of these three compounds. In contrast, chlorogenic acid, the main compound fished by MNPs@TYR possesses a very weak TYR inhibitory activity [32]. These results demonstrate that the cellulose chromatography paper-based ligand fishing method developed in this work is more reliable for screening of TYR inhibitors than the one based on MNPs@TYR.

### 2.6. Enzyme Kinetic Study

**Figure 5:**
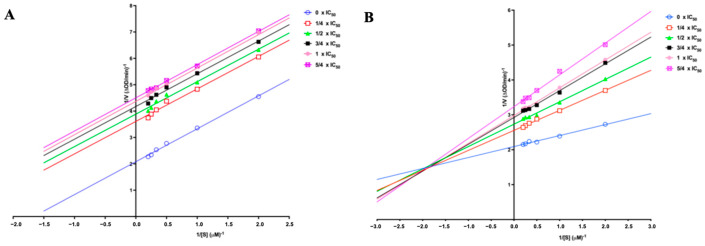
Lineweaver–Burk plots for the inhibition of TYR by (**A**) **1** and (**B**) **3** at different concentrations.

The inhibition type of compounds **1** and **3** against TYR was investigated by Lineweaver−Burk plots, as shown in Figure 5. The x-axis represents the reciprocal of substrate concentrations and the y-axis stands for the reciprocal of reaction rates. The parallel lines observed in the Lineweaver−Burk plots for compound **1** suggest that it exhibits uncompetitive inhibition on TYR, which means compound **1** may bind to the enzyme–substrate complex rather than the free enzyme. In contrast, the intersection of the Lineweaver−Burk plot lines for compound **3** in the upper-left quadrant implies a mixed-type inhibitory effect of the compound, which is characterized by its ability to form complexes with both the enzyme–substrate pair and the enzyme alone.

### 2.7. Molecular Docking

**Figure 6:**
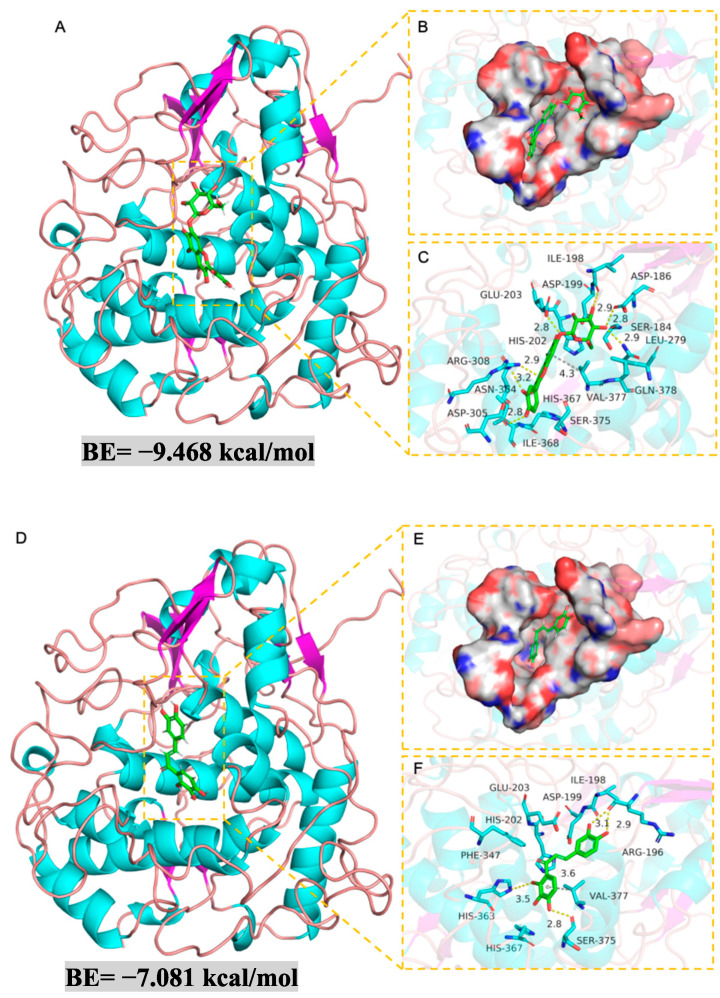
Molecular docking simulations of TYR with compounds **1** and **3**. Three-dimensional docking modes of (**A**) **1** and (**D**) **3** with TYR. The surface of docking modes of (**B**) **1** and (**E**) **3** with TYR. The details of binding modes of (**C**) **1** and (**F**) **3** with TYR. The backbone of protein was rendered in a tube and colored green. Yellow and gray dash lines represent the hydrogen bond and π-stacking, respectively.

Molecular docking was carried out to investigate the inhibition mechanism of compounds. The complexes formed between the protein and small molecules were subjected to Pymol 2.1 for visualization and interpretation. As shown in Figure 6, compounds **1** and **3** can bind to TYR by the molecular interactions including the hydrogen bond, hydrophobic interaction, and π-π stacking. Both compounds displayed high affinity with binding energies below −6 kcal/mol, i.e., −9.468 kcal/mol and −7.081 kcal/mol, respectively. Quercetagetin-7-*O*-glucoside (**1**) establishes multiple hydrogen bonds with amino acids Asp-186, Gln-378, Arg-308, Asp-305, Asn-364, and Ile-198 at the active site of the TYR protein, making significant contributions to anchoring it to the protein’s active site. Additionally, it forms a strong π-π conjugation interaction with Val-377, indicating good compatibility with the protein. Okanin (**3**) establishes hydrogen bonds with Ile-198, Seer-375, and His-363, along with a conjugated interaction with Val-377 residue. These interactions collectively contribute to the formation of a strong complex between the protein and okanin. Overall, both compounds exhibit excellent compatibility with TYR, forming stable complexes.

### 2.8. Inhibition of the Intracellular TYR Activity and Melanin Synthesis

**Figure 7:**
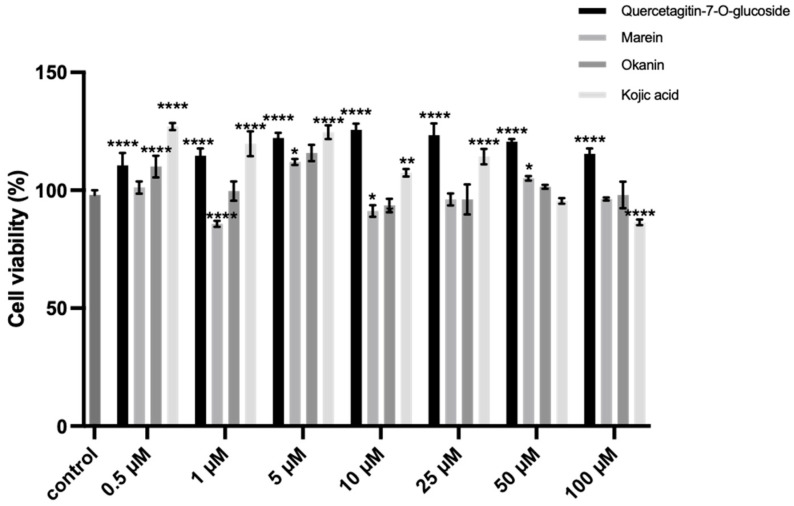
Toxicity of compound **1**–**3** against B16 cells. Kojic acid was used as a positive control. Data (cell viability) are expressed as means ± SD (* *p* < 0.05, ** *p* < 0.01, and **** *p* < 0.0001 compared with the model group).

**Figure 8:**
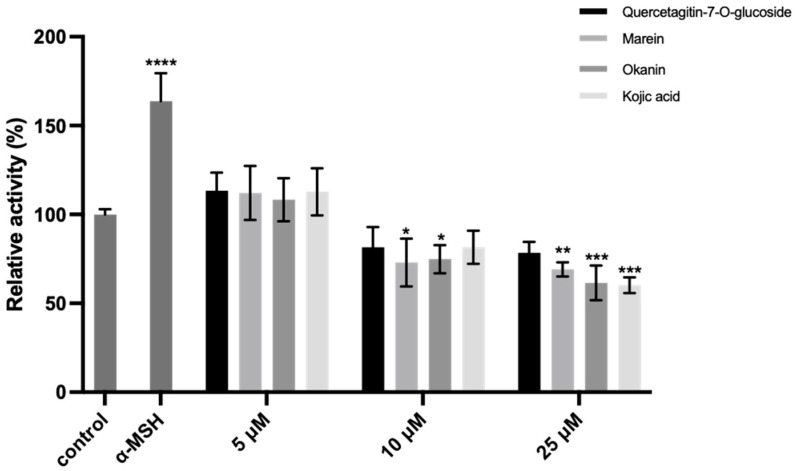
Intracellular TYR inhibition activity of compounds **1–3** at different concentrations. Data (relative activity) are expressed as means ± SD (* *p* < 0.05, ** *p* < 0.01, *** *p* < 0.001, and **** *p* < 0.0001 compared with the model group).

Melanoma B16 cells were first incubated with various concentrations of compounds **1**–**3** as well as kojic acid for 24 h, and the cell viability was measured using the CCK-8 assay. A comparison was made with the blank control group to determine at what concentration these compounds exhibited cytotoxicity. As shown in Figure 7, our compounds did not induce cell death or morphological abnormalities within the selected concentrations. However, the cell viability markedly decreased to 85.77% when the concentration of marein (**2**) was set at 1 µM. The intracellular TYR inhibitory effect of compounds **1–3** (5, 10, and 25 μM) was assessed on the α-MSH (α-Melanocyte-stimulating hormone)-stimulated melanoma B16 cells. As shown in Figure 8, it was found that all the compounds could diminish the TYR activity in a dose-dependent manner, while compound **2** showed the highest inhibition of 59.24% at 25 μM when kojic acid was 65.56%.

**Figure 9:**
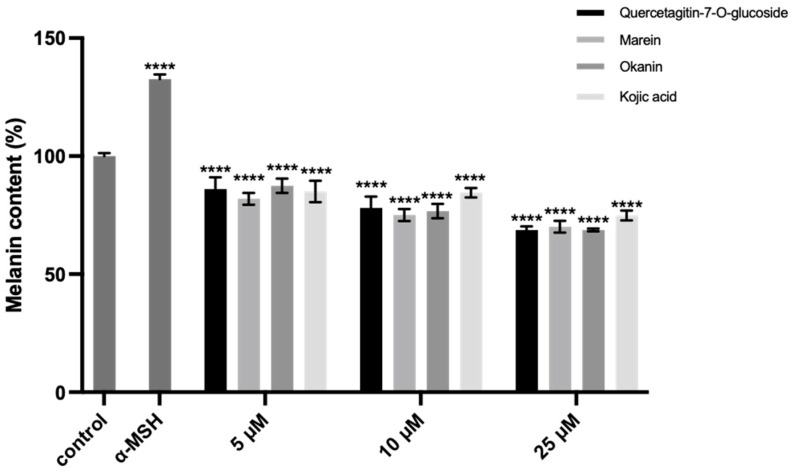
Intracellular melanin reduction activity of compounds **1–3** at different concentrations. Data (melanin content) are expressed as means ± SD (**** *p* < 0.0001 compared with the model group).

Furthermore, the influence of compounds **1**–**3** on the production of melanin in melanoma B16 cells was assayed. It is interesting that despite the weak in vitro TYR inhibition of marein, all three compounds could reduce the melanin levels in the melanoma B16 cells to 72.74%, 73.49%, and 72.71% at 25 μM, comparable to 75.89% of kojic acid when the model group was 129.87% (Figure 9).

## 3. Materials and Methods

### 3.1. Materials and Reagents

Dopamine hydrochloride was obtained from J&K Scientific (Beijing, China). Mushroom Tyrosinase (500 U/mg) was purchased from Puxitang Biotechnology Co., Ltd. (Beijing, China). Whatman cellulose chromatography paper (3 MM Chr3030-704) was purchased from Cytiva Life Sciences (Marlborough, MA, USA). Quercetagetin-7-*O*-glucoside and okanin were purchased from Desite Biotechnology Co., Ltd. (Chengdu, China). Marein was purchased from Push Biotechnology Co., Ltd. (Chengdu, China). The Dulbecco’s modified Eagle’s medium (DMEM) was purchased from Biological Industries (Kibbutz Beit HaEmek, Israel). The melanoma B16 cells were purchased from Shanghai Cell Bank, Chinese Academy of Sciences (Shanghai, China). Cell Counting Kit-8 (CCK-8) was purchased from ProteinTech Group (Chicago, IL, USA). HPLC-grade methanol was supplied by JT Technology (Beijing, China). Ultrapure water was prepared by a UP water purification system (Chengdu, China). Unless otherwise specified, chemicals and solvents were purchased from Kelong Chemical Reagents Factory (Chengdu, China).

### 3.2. Immobilization of TYR on Cellulose Chromatography Paper and Characterization

After the optimization of enzyme immobilization conditions, the cellulose chromatography paper was first cut into circular discs with a diameter of 6 cm and immersed in ultrapure water to be agitated for 20 min for cleaning. Second, the discs were pretreated in a 2% HCl solution under mild agitation for 30 min to expose their microstructure on the surface, followed by seven rinses with ultrapure water for neutralization. Third, we utilized the self-polymerization of dopamine to coat cellulose chromatography paper with a polydopamine film by immersing the cellulose chromatography paper in 1.0 mg/mL of dopamine solution and shaking for 4 h at 30 °C under alkaline conditions (pH 8). The polydopamine-coated cellulose chromatography paper was then taken out and washed seven times with ultrapure water. Dopamine catechol oxidizes to quinone in mild alkaline conditions, which reacts with other catechols or quinones to form polymerized dopamine [33]. The wet CP@DA was blotted with another piece of new dry cellulose chromatography paper, dried at 35 °C, and then immersed in TYR solution (0.1 mg/mL) for 2 h for enzyme immobilization. The polydopamine on the surface facilitated Michael addition and Schiff base reactions with sulfhydryl and amino groups [34]. Finally, the obtained CP@DA@TYR were washed thrice with Tris-HCl (10 mM, pH 7.2), blotted with a fresh cellulose chromatography paper before being dried at 35 °C, and stored at −20 °C.

The surface morphologies of cellulose chromatography paper and CP@DA@TYR were observed by scanning electron microscopy (Thermo Fisher, Waltham, MA, USA).

After the immobilization of the enzyme was complete, the residual enzyme in the solution was quantified to determine the amount of enzyme immobilized on the cellulose chromatography paper. First, serial dilutions of bovine serum albumin (BSA) were prepared from a stock solution of 1 mg/mL to achieve final concentrations of 0.1, 0.08, 0.06, 0.04, 0.02, and 0 mg/mL. Subsequently, 100 μL of each BSA dilution was added to 500 μL of Coomassie Brilliant Blue G-250 dye reagent, and the absorbance of each BSA–dye mixture was measured at 595 nm using a microplate reader. These data were used to generate a standard curve of BSA concentration versus absorbance. Next, six discs of cellulose chromatography paper were immersed in 2 mL of a 0.1 mg/mL TYR solution and shaken for 30 min. Afterward, the cellulose chromatography paper discs were taken out and washed thrice with 2 mL of Tris-HCl buffer solution to remove any unbound enzyme. The washing solutions were then combined with the initial TYR solution. Following this, 100 μL of the combined solution was added to 500 μL of Coomassie Brilliant Blue G-250 dye reagent, and the absorbance was measured at 595 nm to determine the concentration of unbound TYR, using the previously generated BSA standard curve.

### 3.3. Ligand Fishing of the TYR Inhibitors

In the preliminary experiment, we compared the fishing results of *C. tinctoria* from CP@DA@TYR with those from TYR immobilized on magnetic nanoparticles (MNPs@TYR), which we prepared previously [21]. By comparing the TYR inhibitory activity of the mixture of fished compounds (denoted as S5) obtained via two different angling methods (Table S1), we found that CP@DA@TYR was worth investigating for the next screening process.

The procedure for ligand fishing of the TYR inhibitors was performed as follows. A total of 2 mg of the extract was dissolved in 2 mL of Tris-HCl solution and recorded as S0. The CP@DA@TYR with 6 mm in diameter was added into S0 to incubate under gentle agitation for 30 min at 37 °C. Then, the CP@DA@TYR was taken out and washed thrice to eliminate compounds that were adsorbed non-specifically. Finally, the specifically adsorbed ligands were eluted from the CP@DA@TYR using 0.5 mL 50% acetonitrile and recorded as S5. In the meantime, CP@DA in place of CP@DA@TYR was used to serve as the blank control, and the corresponding S5 was recorded as BS5.

### 3.4. Selection of the Buffer System

Different buffer systems were employed for ligand fishing to observe their impact on the performance of the ligand fishing.

### 3.5. Extraction and Isolation of the TYR Inhibitors

The flowers of C. tinctoria were collected in Urumqi City of Xinjiang Uyghur Autonomous Region in July 2023 and identified by Dr. Yong-Mei Zhang at Chengdu Institute of Biology, the Chinese Academy of Sciences, where a voucher specimen (No. 2023-01) was deposited.

The dry flowers (2.0 kg) were powdered and refluxed three times using 80% methanol (10 L × 3), and the combined extracts were evaporated to dryness to give 208 g of crude extract, which was then suspended in H_2_O (1 L) to be sequentially partitioned with petroleum ether (PE, 1 L × 3), ethyl acetate (EtOAc, 1 L × 3), and n-butanol (BuOH, 1 L × 3), respectively. Following HPLC analysis and ligand fishing of each extract fraction, it was determined that all three target compounds were present in the EtOAc fraction (Figure S4). The obtained EtOAc fraction (25 g) was separated by a silica gel column chromatography (200–300 mesh), eluted with gradient CH2Cl2/MeOH (8/1 to 0/1, *v*/*v*) to seven fractions (denoted as Frs. 1–7).

The HPLC analysis was conducted using a Shimadzu (Tokyo, Japan) LC-20AD HPLC system equipped with an SPD-20A UV-visible detector and a reverse-phase Kromasil 100-5-C18 column (4.6 × 250 mm, 5 μm, AkzoNobel, Arlöv, Sweden) under the specified conditions: solvent A was HPLC-grade methanol and B was ultrapure water containing 0.1% formic acid, respectively; the flow rate was 0.8 mL/min; 30–100% A at 0–35 min, 100% A at 35–40 min; the injection volume was 20 μL; column temperature was 35 °C; the detection wavelength was 280 nm. Semi-preparative HPLC was conducted on the Shimadzu LC-20AD system but with a COSMOSIL C18 column (10 × 250 mm, 5 μm, Nacalai Tesque Corporation, Kyoto, Japan), at room temperature.

Among all the seven fractions, Fr. 4, 6, and 7 exhibited the strongest TYR inhibitory effect (43.56%, 39.18%, and 44.87%, Table S1) by in vitro colorimetric assay. Interestingly, only those three gave positive results in the ligand fishing experiment, which is consistent with the enzymatic assay. As depicted in the HPLC chromatograms of the three S5s (Figure S5), compound **3** at Rt 18.2 min was found in S5 of Fr. 4, compound **2** at Rt 14.2 min in S5 of Fr. 6, and compound **1** at Rt 12.1 min in S5 of Fr. 7. Therefore, these ligands were targeted for isolation as follows. Fr. 4 was separated by a silica gel CC eluted with CH_2_Cl_2_/MeOH (8/1 to 0/1, *v*/*v*) to obtain five fractions (Fr. 4-1~Fr. 4-5), among which Fr. 4-3 was purified by semi-preparative HPLC (48% MeOH) to obtain compound **3**. Fr. 6 was separated by CC on Sephadex LH-20 eluted with MeOH followed by semi-preparative HPLC (45% MeOH) to afford compound **2**. Fr.7 was separated by a CC on silica gel with CH_2_Cl_2_/MeOH (6/1 to 0/1, *v*/*v*), followed by semi-preparative HPLC (42% MeOH) to afford compound **1**.

### 3.6. Structural Identification of the TYR Ligands by UPLC-MS

UPLC-MS analysis was carried out on an ACQUITY UPLC I-Class/Vion IMS-QTOF high-resolution LC-MS system (Waters, Milford, MA, USA). The chromatographic analysis was achieved on a Kromasil 100-5-C18 column (4.6 × 250 mm, 5 μm, AkzoNoble, Sweden), and the elution conditions were as follows: solvent A was methanol and B was water containing 0.1% formic acid, respectively; elution gradient was 30–90% A at 0–30 min; the injection volume was 10.0 μL; the detection wavelength was 280 nm. For MS condition, the quadrupole mass analyzer scanned in both positive and negative ion modes; capillary voltage was 3.0 kV in the positive ion mode and 2.5 kV in the negative ion mode; scan range was 50–2000 *m*/*z*; source temperature was 120 °C; desolvation temperature was 450 °C; cone gas was 50 L/h; and desolvation gas was 800 L/h.

### 3.7. Quantification of the TYR Ligands

A 10 mg/mL stock solution was prepared and diluted with methanol into different concentrations of standard solutions from 0.3 to 80 μg/mL and then analyzed by HPLC-UV with the following elution conditions: 40% A at 0−17 min, 100% A at 17−25 min; 45% A at 0−17 min, 100% A at 17−25 min; 52% A at 0−17 min, and 100% A at 17−25 min. Apart from this, the other analytical conditions remained the same as indicated in the above. Calibration curves were established by correlating the integrated areas of chromatographic peaks (y-axis) with the concentrations of the standards (x-axis).

### 3.8. TYR Inhibition Assay

The TYR inhibitory activity of the obtained compounds was assessed using a traditional colorimetric assay with kojic acid as a positive control. Initially, different concentrations of the test sample and 50 U/mL of TYR were prepared with Tris-HCl (10 mM, pH 7.2). Then, 50 μL of tested sample and 50 μL of TYR were mixed and incubated for 10 min at 30 °C in a 96-well plate before 100 μL of L-Dopa (4 mM) was added and incubated for 20 min at 30 °C. Finally, the UV absorbance of the mixture solution was measured at 475 nm by the MULTISKAN GO spectrophotometric plate reader (Thermo Scientific, Waltham, MA, USA). The inhibitory rate (%) was calculated as follows:

(1) $$\mathrm{Inhibition}\;\mathrm{rate}\;(\%)=(1-\frac{A-B}{C-D})\times 100\%$$

where A, B, C, and D are the optical density values (ODs) of the test sample (includes the sample, enzyme, and L-Dopa), sample control (includes sample and L-Dopa), control (includes enzyme and L-Dopa), and blank control (L-Dopa only), respectively. The half maximal inhibitory concentration (IC_50_ values) of each compound was calculated by GraphPad Prism, version 10.0.2.

### 3.9. Enzymatic Kinetic Study

Since marein (**2**) exhibited a weak inhibitory effect, we carried out the enzymatic kinetic study only on quercetagetin-7-*O*-glucoside (**1**) and okanin (**3**) to explore the type of inhibition. The Lineweaver–Burk plot was established through a series of concentrations of L-Dopa (0.5, 1, 2, 3, 4, and 5 mM) at six concentrations of the samples (0, 1/4 × IC_50_, 1/2 × IC_50_, 3/4 × IC_50_, 1 × IC_50_, and 5/4 × IC_50_) while TYR was kept at 50 U/mL. The enzymatic reaction rate was observed for 12 min after starting. The kinetic parameters were calculated as follows:

(2) $$\frac{1}{\nu }=\frac{K_{m}}{V_{max}}\left(1+\frac{\left[I\right]}{K_{i}}\right)\frac{1}{\left[S\right]}+\frac{1}{V_{max}}$$

where K_i_ is the free enzyme inhibition constant; K_m_ is the Michaelis–Menten constant; [S] is the concentration of the substrate; [I] is the concentrations of the inhibitor; V is the reaction rate; and V_max_ is the maximum reaction rate, respectively.

### 3.10. Molecular Docking

Molecular docking was used to investigate the inhibitory mechanism of the inhibitors against TYR.

Small molecule structures from PubChem (https://pubchem.ncbi.nlm.nih.gov/, accessed on 21 January 2024) were optimized with Chem3D and imported to Schrödinger 2019 for docking database creation. Procedures included hydrogenation, structural optimization, and energy minimization. Tyrosinase (TYR, ID: P14679) structure was sourced from UniProt (https://www.uniprot.org/, accessed on 21 January 2024) and processed via Maestro 11.9 on Schrödinger. This included dehydrating, hydrogen addition, bond repair, peptide segment correction, energy minimization, and structure optimization. Molecular docking, conducted using Schrödinger’s Glide and LigPrep for protein and ligand preparation, respectively, involved receptor optimization with the OPLS3e force field and binding site prediction within a 10 Å × 10 Å × 10 Å cube, employing the standard precision method for evaluation. Compound–protein interactions were analyzed by the interaction forces, with docking scores assessed for potential activity. 

### 3.11. In Vitro TYR Inhibition Activity

#### 3.11.1. Cell Culture

The melanoma B16 cells (from Shanghai Cell Bank, Chinese Academy of Sciences) were cultured in DMEM (contains 10% FBS and 1% penicillin-streptomycin) and maintained at 37 °C with 5% CO_2_ in a humid atmosphere.

#### 3.11.2. Cytotoxicity Assay

The cytotoxicity of compounds **1**–**3** to the melanoma B16 cell model, with kojic acid as a positive control, was conducted in vitro using the CCK-8 method. A total of 1 × 10^4^ cells were seeded into each well in a 96-well plate and incubated for 24 h. The cells were then treated with different concentrations of test samples (0.5, 1, 5, 10, 25, 50, and 100 μM) for 24 h after the initial culture medium was removed. Afterward, 10 μL of CCK-8 solution was added into each well and incubated for 1 h before the UV absorbance was measured at 450 nm.

#### 3.11.3. Intracellular TYR Inhibition Activity Assay

After the selection of the appropriate concentration range that exhibits no cytotoxicity, the intracellular TYR inhibitory activity was conducted, with kojic acid serving as a positive control. A total of 1 × 10^5^ cells were seeded into each well in 6-well plates. After incubation for 24 h, the medium was replaced by a fresh one containing 1 μM α-MSH (α-Melanocyte-stimulating hormone) together with different concentrations of the test compound (5, 10, and 25 μM) to be incubated for another 48 h. The culture medium was removed, and the cells were washed twice with Tris-HCl before adding 300 μL of 1% Triton X-100 for cell scraping. Then, L-Dopa was added and incubated for 30 min. Finally, 100 μL of the resulting mixture was taken from each sample in triplicate in a 96-well plate, and UV absorbance was measured at 475 nm.

The inhibitory rate (%) was calculated as follows:

(3) $$\mathrm{Inhibition}\;\mathrm{rate}\;(\%)=(1-\frac{A}{C})\times 100\%$$

where A and C are the ODs of the test group (includes 1 μM α-MSH with different concentrations of test compounds) and model group (1 μM α-MSH only), respectively.

#### 3.11.4. Quantification of Intracellular Melanin Content

The intracellular melanin content was quantified by the previously reported method with minor modifications [35]. The cells were cultured as described in the first part of Section 3.11.3; however, when treated with 1 μM of α-MSH with the test compounds (**1**–**3**), the time for incubation was 72 h rather than 48 h. Then, the supernatant was discarded and the cells adhering to the walls of the wells were trypsinized, and transferred to Eppendorf tubes to centrifuge at 4000 rpm for 5 min at 10 °C. After discarding the supernatant, the cell pellets were lysed with 1 M NaOH solution containing 10% DMSO for 1 h at 80 °C and then centrifuged at 12,000 rpm for 15 min at 10 °C. The UV absorbance of the clear supernatant was measured at 405 nm to assess melanin content.

### 3.12. Statistical Analysis

The results were derived from three independent experiments and are presented as the mean ± standard deviation (SD). Statistical significance was assessed using two-way ANOVA. Differences were deemed statistically significant when *p* < 0.05. All data analyses and graphical representations were performed using GraphPad Prism software 10.0.2, ensuring a thorough evaluation of the experimental outcomes.

## 4. Conclusions

In conclusion, by immobilizing the enzyme on Whatman cellulose chromatography paper as a fishing bait, we developed a cheap, convenient, and reliable ligand fishing method for screening of tyrosinase inhibitors from complex herbal extract. Three new TYR inhibitors were found in flowers of *C. tinctoria*, i.e., quercetagetin-7-*O*-glucoside (**1**), marein (**2**), and okanin (**3**). The kinetics study identified the mixed-type inhibition of **1** and uncompetitive inhibition of **3** toward TYR, and molecular docking revealed interaction mechanisms between the compounds and the enzyme. All three compounds significantly inhibit the activity of intracellular TYR and the production of melanin in melanoma B16 cells. To the best of our knowledge, this is the first report on the TYR inhibitory effect of those three flavonoids, as well as the identification of skin-lightening components present in the flowers of *C. tinctoria*. It is of high significance for better utilization of this plant in the fields of skin care and the health food industry.

## Data Availability

Data are contained within the article and Supplementary Material.

## Supplementary Materials

The following supporting information can be downloaded at: https://www.mdpi.com/article/10.3390/molecules29174018/s1, Figure S1: Schematic illustration of the workflow for TYR inhibitor screening from *Coreopsis tinctoria*; Figure S2: Scanning electron microscopy (SEM) images of CP (A) and CP@DA@TYR (B) at varying levels of magnification; Figure S3: UPLC-MS analysis of S5; Figure S4: HPLC chromatograms of different extractions; Figure S5: HPLC comparison of S0 and S5 from Frs.1–7; Table S1: Inhibitory effect of samples on TYR.
