# Supplementary material for: Fast Screening of Tyrosinase Inhibitors in Coreopsis tinctoria Nutt. by Ligand Fishing Based on Paper-Immobilized Tyrosinase
Source: Molecules. 2024 Aug 25;29(17):4018. doi: 10.3390/molecules29174018 (PMC11397727; doi:10.3390/molecules29174018)
Supplement: Supplementary file 1 [file molecules-29-04018-s001.zip › molecules-3144074-supplementary.pdf]

Supporting Information

Fast screening of tyrosinase inhibitors in *Coreopsis tinctoria* Nutt. by ligand fishing based on paper-immobilized tyrosinase

Ayzohra Ablat [1, 3, #], Ming-Jie Li [2, #], Xiao-Rui Zhai [1, 3], Yuan Wang [2], Xiao-Lin Bai [1], Peng Shu [2, *], and Xun Liao[1, *]

1. Chengdu Institute of Biology, Chinese Academy of Sciences, Chengdu 610041, China
2. HBN Research Institute and Biological Laboratory, Shenzhen Hujia Technology Co., Ltd., Shenzhen 518000, China
3. University of Chinese Academy of Sciences, Beijing 100049, PR China

# These authors made the same contribution to this work.

Author contact details:

Ayzohra Ablat: ayi@cib.ac.cn

Ming-Jie Li: limingjie@hbn.cn

Xiao-Rui Zhai: zhaixr@cib.ac.cn

Yuan Wang: wangyuan@hbn.cn

Xiao-Lin Bai: baixl@cib.ac.cn

[*] Corresponding authors:

Dr. Xun Liao, Email address: liaoxun@cib.ac.cn; Tel: +86-28-82890402

Dr. Peng Shu, Email address: shupeng20@mails.ucas.ac.cn; Tel: +86-755-86562982

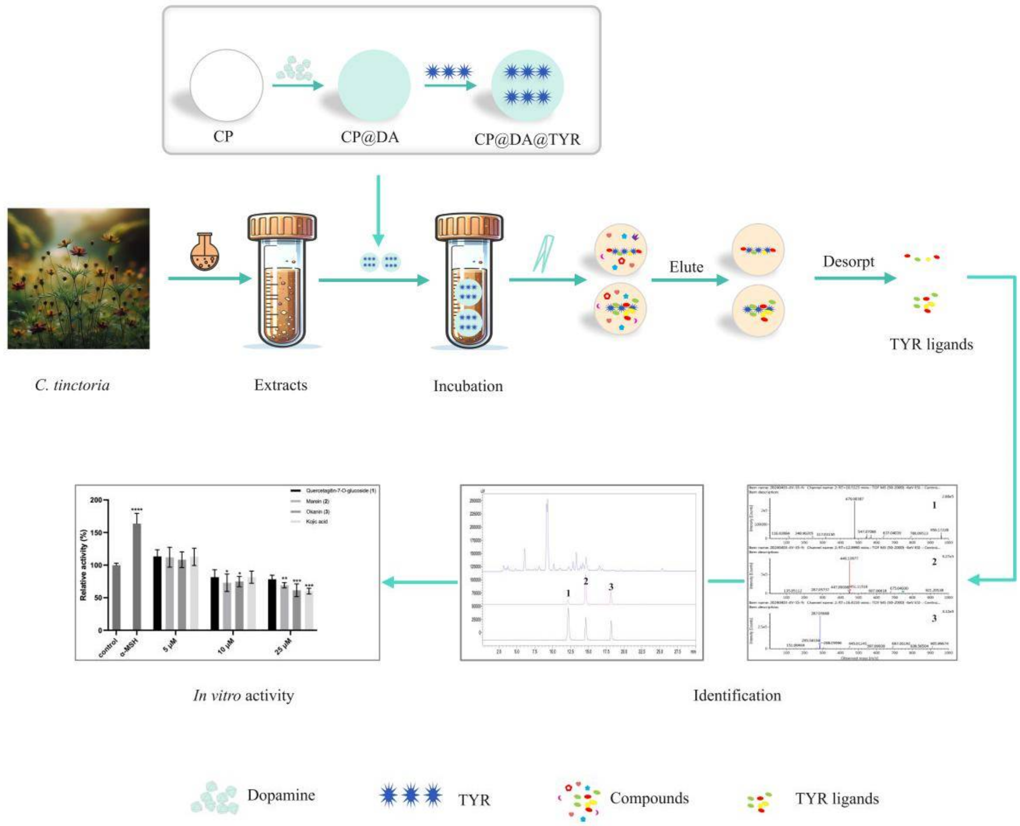

Figure S1. Schematic illustration of the workflow for TYR inhibitor screening from *Coreopsis tinctoria*.

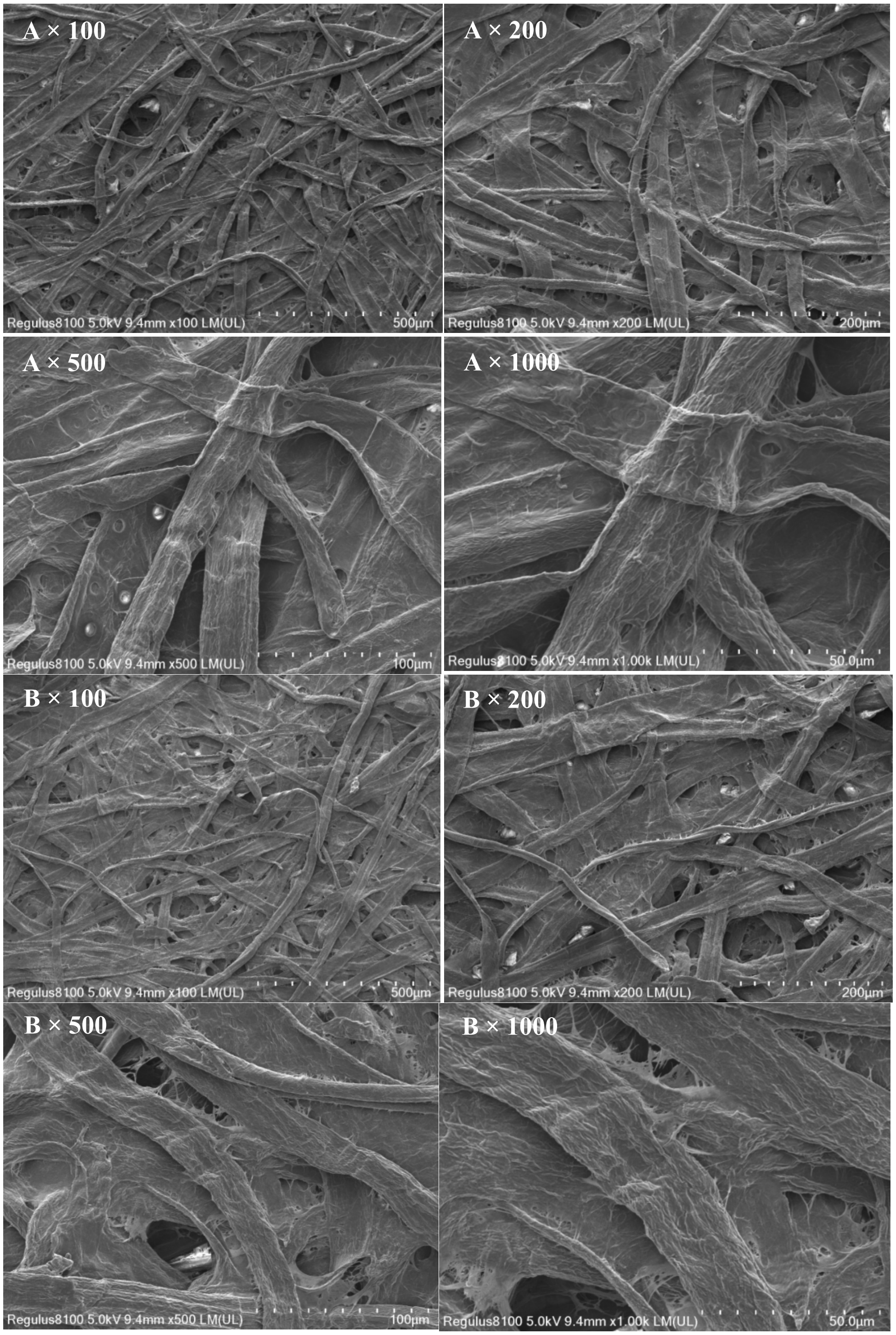

Figure S2. Scanning electron microscopy (SEM) images of CP (A) and CP@DA@TYR (B) at varying levels of magnification.

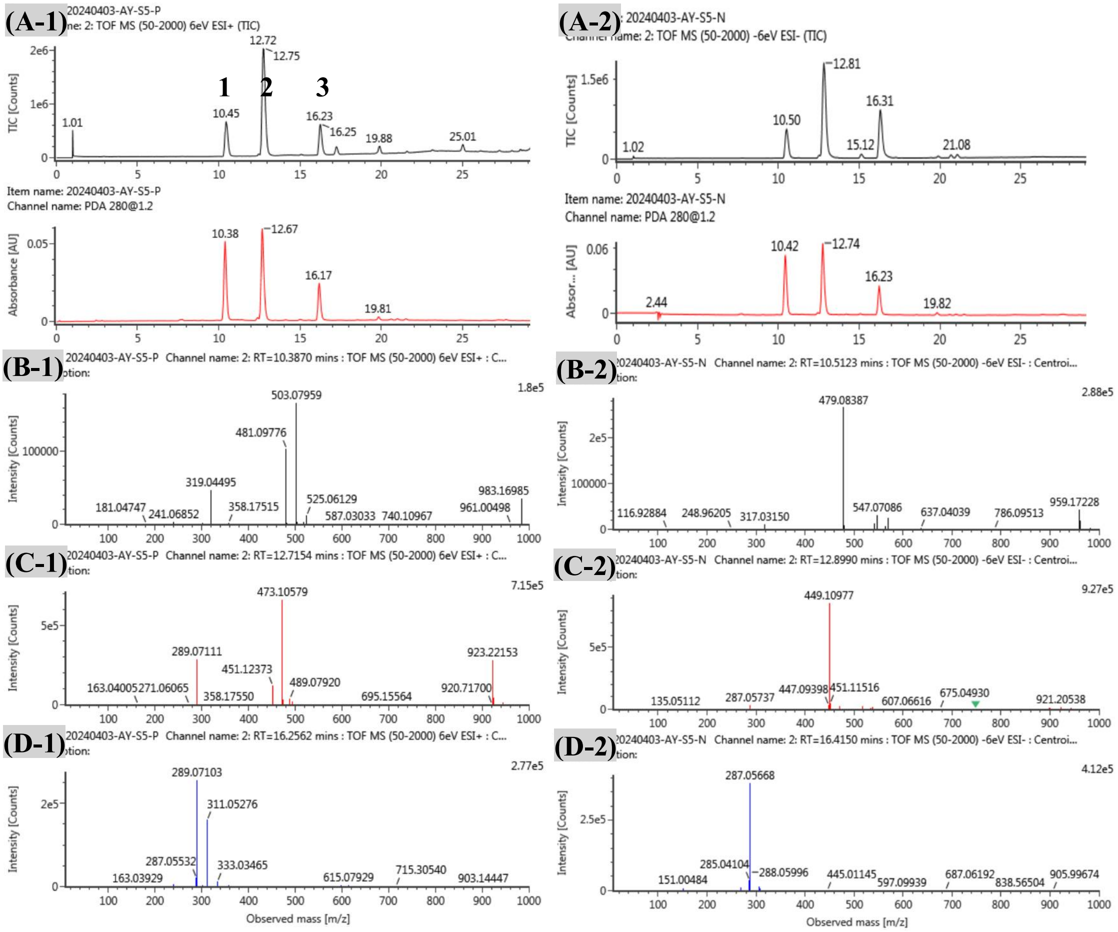

Figure S3. UPLC-MS analysis of S5. A: total ion chromatograms of S5 (A-1 for positive ion mode and A-2 for negative ion mode). B-D: mass spectra of quercetagetin-7-*O*-glucoside (**1**), marein (**2**), and okanin (**3**). B-1, C-1, and D-1 are positive ion mode, B-2, C-2 and D-2 are negative ion mode.

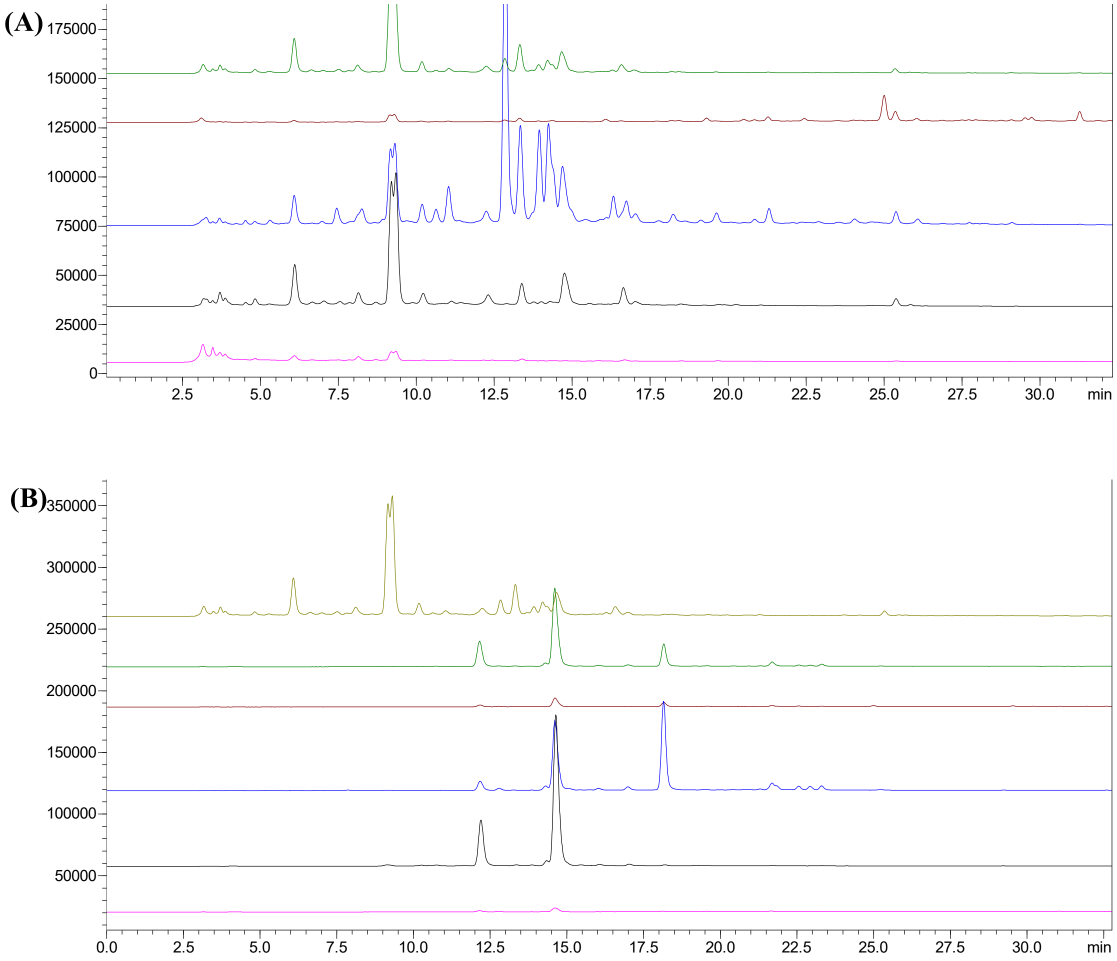

Figure S4. (A) HPLC chromatograms of different extractions. From top to bottom: S0, PE extraction, EA extraction, BuOH extraction, and H_2O extraction. (B) HPLC chromatogram of S5s. From top to bottom: S0, S5, PE-S5, EA-S5, BuOH-S5, and H_2O-S5.

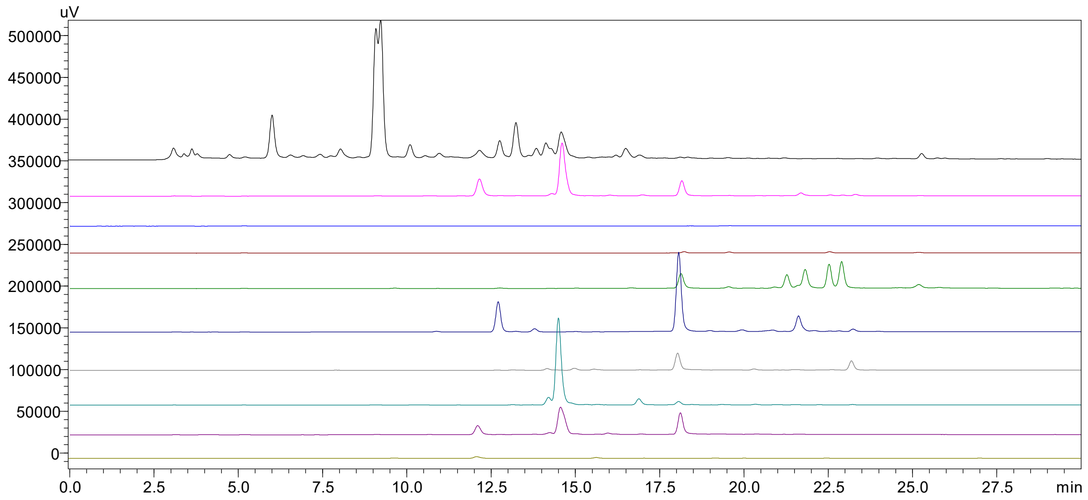

Figure S5. HPLC comparison of S0 and S5 from Frs.1-7. From top to bottom: S0, S5, Fr.1-S5, Fr.2-S5, Fr.3-S5, Fr.4-S5, Fr.5-S5, Fr.6-S5, Fr.7-S5, and BS5.

Table S1. Inhibitory effect of samples on TYR.

Samples	Inhibition rate (%)
C. tinctoria crude extract	35.43
MNPs@TYR-S5	26.24
CP@DA@TYR-S5	38.67
Fr.1	7.02
Fr.2	2.11
Fr.3	18.23
Fr.4	43.56
Fr.5	23.08
Fr.6	39.18
Fr.7	44.87
kojic acid	82.11

Values are expressed as mean ± standard deviation (SD) (n = 3). 1 mg/mL for S0 and S5, 0.1 mg/mL for Frs.1-7, and kojic acid.
